# An evaluation of Brazil’s surveillance and prophylaxis of canine rabies between 2008 and 2017

**DOI:** 10.1371/journal.pntd.0007564

**Published:** 2019-08-05

**Authors:** Julio A. Benavides, Jane Megid, Aline Campos, Silene Rocha, Marco A. N. Vigilato, Katie Hampson

**Affiliations:** 1 Departamento de Ecología y Biodiversidad, Facultad de Ciencias de la Vida, Universidad Andrés Bello, Santiago, Chile; 2 UNESP - Faculdade de Medicina Veterinária e Zootecnia, Departamento De Higiene Veterinária e Saúde Pública, Botucatu, São Paulo, Brazil; 3 Institute of Biodiversity, Animal Health and Comparative Medicine, University of Glasgow, Glasgow, United Kingdom; 4 Centro de Investigación para la Sustentabilidad, Facultad de Ciencias de la Vida, Universidad Andrés Bello, Santiago, Chile; 5 Programa Estadual de Controle e Profilaxia da Raiva, Health Secretary of Rio Grande do Sul, Porto Alegre, Brazil; 6 Pan-American Health Organization, Veterinary Public Health Unit – PANAFTOSA, Rio de Janeiro, Brazil; US Department of Agriculture, UNITED STATES

## Abstract

An effective surveillance system is critical for the elimination of canine rabies in Latin America. Brazil has made substantial progress towards canine rabies elimination, but outbreaks still occurred in the last decade in two states. Brazil uses a health information system (SINAN) to record patients seeking post-exposure prophylaxis (PEP) following contact with an animal suspected of having rabies. This study evaluated: (i) whether SINAN can be reliably used for rabies surveillance; (ii) if patients in Brazil are receiving appropriate PEP and (iii) the benefits of implementing the latest World Health Organization (WHO) recommendations on PEP. Analysing SINAN records from 2008 to 2017 reveals an average of 506,148 bite-injury patients/year [range: 437k-545k] in the country, equivalent to an incidence of 255 bite-injuries/100,000 people/year [range: 231–280]. The number of reports of bites from suspect rabid dogs generally increased over time. In most states, records from SINAN indicating a suspect rabid dog do not correlate with confirmed dog rabies cases reported to the Regional Information System for Epidemiological Surveillance of Rabies (SIRVERA) maintained by the Pan American Health Organization (PAHO). Analyses showed that in 2017, only 45% of patients received appropriate PEP as indicated by the Brazilian Ministry of Health guidance. Implementation of the latest WHO guidance using an abridged intradermal post-exposure vaccination regimen including one precautionary dose for dog bites prior to observation would reduce the volume of vaccine required by up to 64%, with potential for annual savings of over USD 6 million from reduced vaccine use. Our results highlight the need to improve the implementation of SINAN, including training of health workers responsible for delivering PEP using an Integrated Bite Case Management approach so that SINAN can serve as a reliable surveillance tool for canine rabies elimination.

## Author summary

Dog-mediated rabies has declined to only a few cases in Latin America over the last decade. Brazil has the largest human and dog population of Latin America. Despite the decline of canine rabies, the country’s public health system still spends millions of dollars annually on half a million patients seeking health care for dog bites. In this study, we analysed a decade of national surveillance data on dog bites. These data suggest that health workers report dog rabies in many states where the disease is likely to be absent, with false positive cases frequently reported into the surveillance system. In addition, only half of patients appear to receive the appropriate rabies post-exposure prophylaxis as recommended by the Ministry of Health. We estimated that Brazil could save up to USD 6 million per annum on vaccine by reducing the number of doses administered during prophylaxis and adopting the intradermal vaccine delivery technique following the latest WHO recommendations. Our study highlights an urgent need for updating health care workers on canine rabies knowledge, prophylaxis and assessment of dog bites to improve prophylaxis provision and surveillance of dog rabies.

## Introduction

Dog-mediated rabies in humans has been reduced by over 99% in Latin America, but 106 cases in dogs were still reported in 2014 [1]. Dog vaccination and prompt administration of post-exposure prophylaxis (PEP) for bite victims are essential to the successful reduction of human cases of rabies across Latin America [2,3]. However, detecting the final cases of rabies and interrupting transmission is difficult and therefore continued precautionary use of PEP is required for persons bitten by dogs that show signs suspicious of rabies [4]. In Latin America, PEP is broadly available and 0.2% of people are referred to health services annually after potential exposure to the risk of rabies (mainly because of bites by dogs), with around 25% of patients receiving free PEP [3]. Control of rabies in dogs is expected to reduce bites by rabid dogs but not necessarily bites by healthy dogs for which patients do not require PEP. Unnecessary and indiscriminate use of PEP strains local and national healthcare budgets, and is a missed opportunity for improving the detection of rabies cases [5,6].

Eliminating dog-mediated rabies requires an efficient and sensitive surveillance system that can detect new, but rare, outbreaks of dog rabies. However, outbreaks of dog rabies are difficult to detect without using targeted methods and community surveillance because of the millions of dogs in Latin America and because rabies incidence is low even when endemic (less than 1% of dogs become infected per annum) [7]. Passive surveillance of dog bite victims seeking help within the health care system can support active surveillance in detecting animal rabies cases [8–10]. Dog rabies can be detected by focusing on bite patients and evaluating the dogs responsible for bites, because dog bites are frequent and unprovoked aggression is one the main clinical signs of this disease. Surveillance of bites can also reveal secondary cases caused by a rabid dog and has potential to reduce unnecessary PEP use [5,8,10,11]. For example, surveillance of dog bites in Haiti identified areas of rabies risk and led to considerable reductions in PEP use [8,11]. Improved surveillance of dog-bite patients can also help to detect the rare but increasing cases in dogs caused by bat rabies in countries such as Brazil [12].

Brazil has one of the largest estimated populations of dogs worldwide and the second largest of the continent after the United States, with around 30–50 million dogs [13,14]. The country has made substantial progress towards the elimination of dog-mediated rabies over the last decades, but cases persist in the state of Maranhão, and a large outbreak recently occurred in Mato Grosso do Sul as a result of incursions from bordering Bolivia [15,16]. Thus, Brazil requires an effective surveillance system to support current dog vaccination campaigns to eliminate rabies from remaining endemic foci. Brazil’s National Health System (SUS) provides universal health coverage to most Brazilians using a decentralized network of public hospitals and health facilities. Since 1998, Brazil has used a national health ‘Information System on Diseases of Compulsory Declaration’ (SINAN) to record all patients seeking medical care in public health facilities following contact with an animal that can transmit rabies. SINAN is a relatively inexpensive tool that records information on patients, the animal contacted and PEP use. The SINAN database records several variables that can be used for rabies surveillance including the severity of the bite, whether the biting animal is suspected for rabies, whether the animal is alive and can be observed for a 10-day period, and the PEP regimen administered to the patient [17,18].

Previous studies have performed descriptive analysis to summarize the SINAN database at national and state level [18–21], but the accuracy of the SINAN database as a tool for the surveillance of rabies and for the appropriate use of PEP has not been evaluated. In Brazil, dog bite incidence was high between 2002 and 2009 with 425 000 bites estimated per year [19,22]. The reduction of canine rabies cases over the last decade in Brazil should cause a concommittant decline in bites from rabid dogs and potential savings in PEP administration, but this has not been realized. Here we examine (i) whether SINAN can be reliably used for rabies surveillance; (ii) if patients in Brazil are receiving appropriate PEP according to Brazilian Ministry of Health (MoH) guidelines and (iii) the benefits of implementing the latest World Health Organization (WHO) recommendations on PEP.

## Methods

We obtained data from the ‘Individual Investigation Reports of Human Anti-rabies Care’ form completed by public health workers in Brazil and submitted to SINAN [17]. This form can be filled by any health professional (doctor, nurse, technician) each time a patient seeks care at a public health facility following an animal bite. Each paper form (available here in portuguese:http://portalsinan.saude.gov.br/images/documentos/Agravos/Atendimento%20Anti-rabico/anti_rabico_v5.pdf) includes 60 fields and is entered into the national electronic system. The SINAN notification (electronic data) of the aggression (bite) must be sent weekly from municipality to state level, and every two weeks from State to Federal level. The municipality has 60 days to finish the investigation and conclude the case [23]. All cases must be concluded [23]. The investigation includes the use and follow-up of PEP after assessment by a doctor or nurse, observation of the potential rabid dog (mainly conducted by the patient) and laboratory testing of dogs considered as ‘rabid’ within the first assessment upon the patient’s arrival. Cases are concluded when PEP is finished, when the patient interrupts PEP (e.g. to complete PEP in another facility), when the outcome of the dog observation period is known or when the laboratory test outcome on a suspicious dog is returned.

We focused on forms completed for dog bites. The form is divided into 5 sections including (i) general data on the health unit, (ii) patient characteristics, (iii) location of patient’s residency and health care unit (e.g. municipality and state), (iv) epidemiological information including the animal responsible for the bite, whether it is considered as suspicious for rabies and the prior PEP history of the patient as well as (v) the PEP recommended and given to the patient and the status of the dog after a potential 10-day observation period. All variables included in our analysis of these SINAN forms are listed in S1 Table of the Supplementary Material. Data were obtained for Brazil from 2008 to 2017 by requesting the data online to the Ministry of Health through the Electronic System of Citizen’s information (e-SIC, https://esic.cgu.gov.br/sistema/site/index.aspx, e-SIC request number: 2564134). Data, which can be requested from the Ministry of Health but cannot be shared publicly, was received as standard database files (.dbf) and analysed using R 3.4.3 [24]. Following the Ministry of Health’s ethical guidelines, we did not receive identifiable data on patients (section ii). Empty fields were assumed to have not been completed and were shown in the analysis as ‘no data available’. We developed an algorithm as a pilot analysis of the data to detect inconsistencies between fields, such as forms simultaneously indicating a dog as ‘dead’ and ‘alive and observable’ in different fields. This indicated that an average of 5% (range: 1–17%) of forms per state within a year contained incongruent data. For these records, we used the most reliable source of data for the analysis that was consistent with other fields in the form. For example, if the responsible dog was declared as ‘dead’ in one field yet ‘alive and observable’ in another, we used a third variable such as the result of the observation period to assign a status as to whether the dog was considered alive or not. We also performed analyses excluding these inconsistent forms, but this did not affect observed temporal nor spatial patterns.

We also obtained data from the Regional Information System for Epidemiological Surveillance of Rabies (SIRVERA) database, where records indicate confirmed rabies cases by species that are submitted by each Brazilian state to PAHO [3]. SIRVERA is the officially recognized rabies surveillance system of the Americas. These data comprise both curated reports that originated from SINAN (but have been verified by each State’s rabies program or the Ministry of Health) and dogs that are reported as dead by veterinarians, health workers or the public and are subsequently confirmed through laboratory diagnosis. Data from SIRVERA are publicly available online: http://sirvera.panaftosa.org.br/.

### Data analysis

#### Temporal and spatial trends of bite incidence and suspect rabid dogs in Brazil

We first explored whether bite incidence, here the number of patients seeking health care after a dog bite per 100,000 habitants, varied between 2008 and 2017 and across states. We calculated bite incidence as the number of completed reports divided by the total human population of each state, extracted from publicly available data from the Brazilian Institute of Geography and Statistics (IBGE) (https://www.ibge.gov.br/). Bite incidence can depend on several factors including dog density within an area and socioeconomic status, with low-income areas typically having higher dog bite incidence [25,26] and lower access to health care [27,28]. Thus, we included a correlation analysis between bite incidence and (i) the average income of each state extracted from the IBGE website for each year and (ii) the number of houses with at least one dog and the percentage of houses with at least one dog estimated in 2013 by the IBGE (https://sidra.ibge.gov.br/tabela/4930) which are, to our knowledge, the only official variables related to dog population in the country. Upon a patient’s arrival to a health center, a health worker interviews the patient and assesses the condition of the dog responsible for the bite before recommending PEP. Each dog is therefore assessed as belonging to one of four categories: ‘healthy’, ‘rabies suspicious’, ‘rabid’ or ‘dead/disappeared’. Using these categories, we calculated the monthly number of bites from dogs classified as ‘suspect’ (all categories except ‘healthy’) in each state by year. Since 10 of 27 states did not enter bite forms in the last three months of 2017 into SINAN, results on bite incidence were compared between all states using data from 2016. All other analyses focusing on percentages of total number of forms included 2017 data.

#### Comparison of suspect rabid dogs recorded in SINAN and SIRVERA

The SINAN database also includes a field on the outcome of a 10-day period of dog observation recommended by health workers if the dog showed signs suspect for rabies or if the bite was considered as severe. At the end of this observation period, each dog was classified to one of five categories: ‘healthy’, ‘dead’, ‘disappeared’, ‘clinical signs of rabies’, or ‘laboratory confirmed rabies’. To assess the accuracy of this risk assessment extracted from SINAN, we compared the monthly number of ‘laboratory confirmed rabies’ records in SINAN for each state with the number of ‘laboratory confirmed’ dog rabies cases reported to SIRVERA. Positive cases in SINAN that did not appear in SIRVERA were considered as ‘false positives’.

#### Estimation of appropriate PEP administration according to the Brazilian MoH

**Fig 1:**
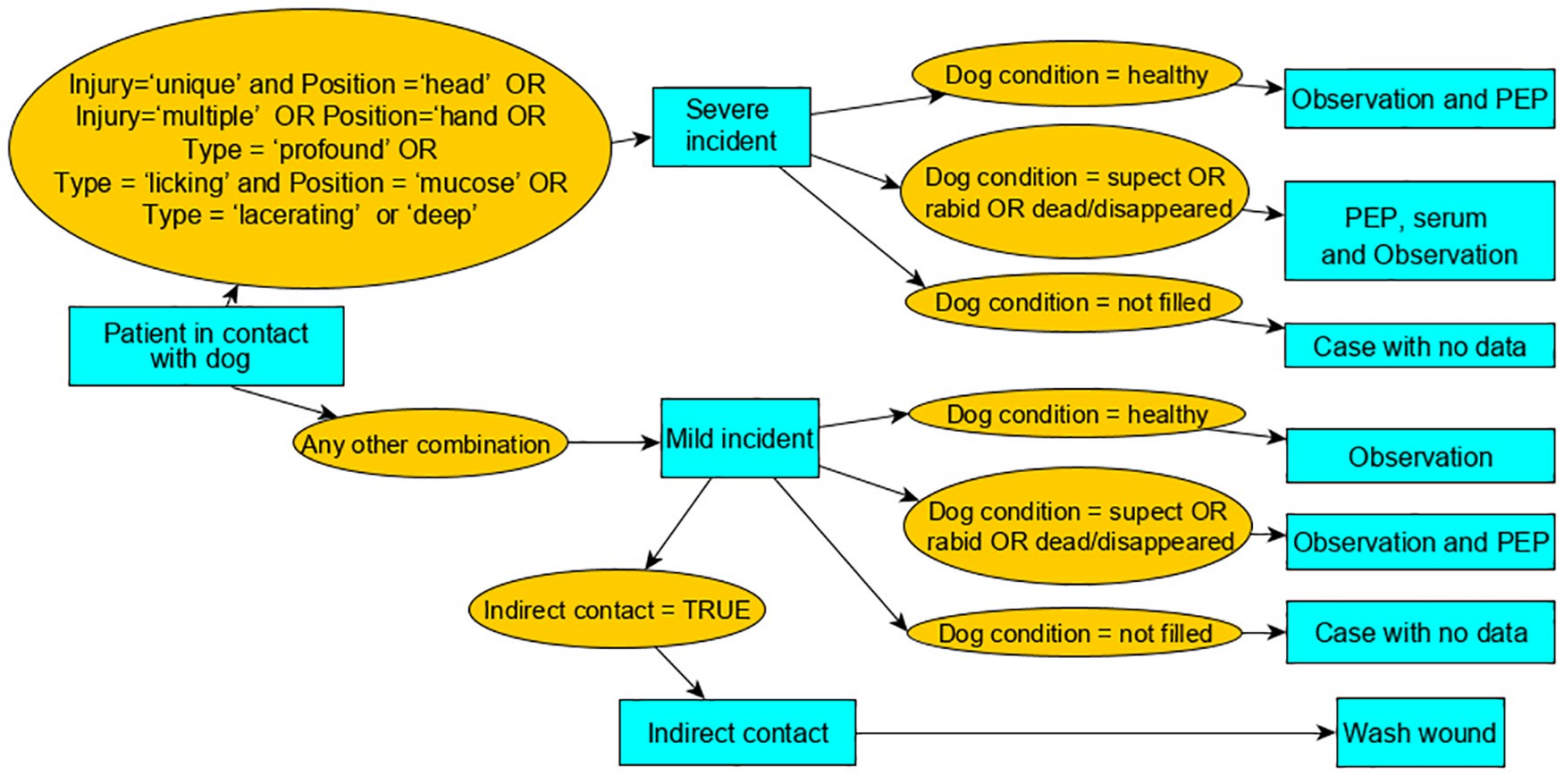
Algorithm used to evaluate the appropriateness of the PEP administered as recorded in each SINAN form according to the guidelines of the Brazilian MoH. Rectangles show the Brazilian MoH’s recommendation (see S2 Table) based on the criteria filled on the SINAN form (circles). For patients requiring PEP and/or serum, the full algorithm included a further step discriminating between individuals that previously received complete PEP at least 90 days prior to the current exposure, in which case they did not need either vaccine or serum. Patients requiring serum and previously receiving a pre-exposure prophylaxis were considered as patients without previous PEP because the form does not include whether their titer levels were >0.5IU/ml, in which case serum is not recommended.

To evaluate if PEP is being effectively delivered in Brazil, we compared the PEP given to each patient recorded in SINAN in 2017 with the appropriate PEP for the same patient according to the Brazilian’s Ministry of Health (MoH) prophylaxis guidelines from 2014, based upon the variables reported in SINAN. The SINAN form includes seven PEP recommendations: ‘Pre-exposure prophylaxis’, ‘No prophylaxis’, ‘Observe the dog for 10 days but no vaccine or serum’, ‘Observe the dog and administer vaccine’, ‘Administer vaccine but no serum’, ‘Administer vaccine and serum’, ‘Re-exposure prophylaxis’. We used an algorithm to calculate ‘appropriate’ PEP considering the risk assessment data from each SINAN form according to the MoH guidelines. The MoH guidelines are based on three criteria: (i) bite severity, (ii) dog status before and after the 10-day period observation and (iii) previous PEP history (vaccination/vaccine titers). Details of these guidelines are available in the Supplementary S2 Table. The algorithm is presented in Fig 1. First, the algorithm differentiates each SINAN form as either a ‘severe’ or ‘mild’ incident according to the MoH criteria (Fig 1 and S2 Table) using three specific criteria: the type of exposure, the position of the exposure, and the injury and type of injury. If an exposure did not fulfil the criteria to be classified as a ‘severe incident’, it was classified as a ‘mild’ incident. We considered forms where the type of exposure was filled as ‘other or ignored’, the ‘position of exposure’ as ‘unknown’, the ‘injury’ as ‘ignored’ or the ‘type of injury’ as ‘ignored’ as mild incidents. These forms accounted only for 2.3% of mild incidents, therefore we suspect that if some were wrongly diagnosed (i.e. were severe), this would not greatly affect our results. Second, the algorithm further discriminated PEP based on whether the dog was ‘healthy’, ‘rabies suspicious’, ‘rabid’ or ‘dead/disappeared’ (Fig 1). Finally, the algorithm separated patients on whether they had received complete PEP previously, which reduced their PEP requirements.

We then compared the ‘administered’ versus ‘appropriate’ PEP, as a percentage of total patient presentations. Further evaluation on appropriate PEP should examine whether the correct number of doses was administered but this information was not completed in the electronic SINAN, and therefore not included in our analysis. Three southern states of Brazil (Rio Grande do Sul, Paraná and Santa Catarina) changed their PEP recommendations to a more conservative use of PEP, recommending ‘10-day of dog observation’ for patients bitten by observable healthy dogs rather than initiating PEP regardless of ‘bite severity’, following their own declaration of dog-rabies freedom in 2017. We did not investigate these changes, since the percentage of health centers implementing these new guidelines was not known.

#### Cost savings under updated PEP guidelines

Recent updates to both the Brazilian PEP guidelines in late 2017 and the WHO position on rabies vaccines and immunoglobulins in early 2018 [29] include more judicious use of PEP and dose-sparing Intradermal (ID) vaccination regimens. We evaluated the potential for cost savings to be made through the implementation of these recommendations by analysing expected PEP costs in 2017 under these changes. We evaluated potential for cost savings in terms of volume of vaccine used, which was then multiplied by a monetary value per ml. We assumed a cost of 10 USD for each dose of 0.5ml vaccine, which is the approximate cost of vaccine in 2018 listed by the Ministry of Health. We calculated PEP costs under 3 scenarios: (i) the 2016 protocol in Brazil comprising 5 doses of vaccine according to the intramuscular (IM) Essen regimen if the dog was considered suspect for rabies and 2 IM doses only if the dog was considered healthy after observation, (ii) as per (i) but with the updated 2017 Brazilian guidelines using the updated IM Essen regimen with 4 doses and (iii) the latest WHO position with 3 ID doses for suspect rabies exposures as a full prophylaxis and 1 precautionary dose only if the dog is healthy and remains so under observation (instead of 2 doses as in the MoH protocol). The latest WHO position recommends a dose-sparing ID regimen [29], which reduces the volume of vaccine required, especially when vials can be shared between patients presenting to facilities on the same day [30,31]. Thus, for scenario three we examined two extremes: the situation whereby vials are not shared between patients (‘no vial sharing’), i.e. one vial is used for each patient visit (that requires 2x0.1 ml injections from a 0.5 ml vial and the remainder is discarded) compared to the situation where vials are shared completely between patients, with the remaining volume used for subsequent patients (‘complete vial sharing’).

#### Ethics statement

This project was approved by the Brazilian Ministry of Health’s Ethics Committee (‘Plataforma Brasil’, CAE # 94081818.0.0000.5411) through the Ethics Committee of the Faculty of Medicine of the São Paulo State University (UNESP) -Botucatu.

## Results

### Temporal and spatial trends of bite incidence and suspect dogs in Brazil from SINAN

**Fig 2:**
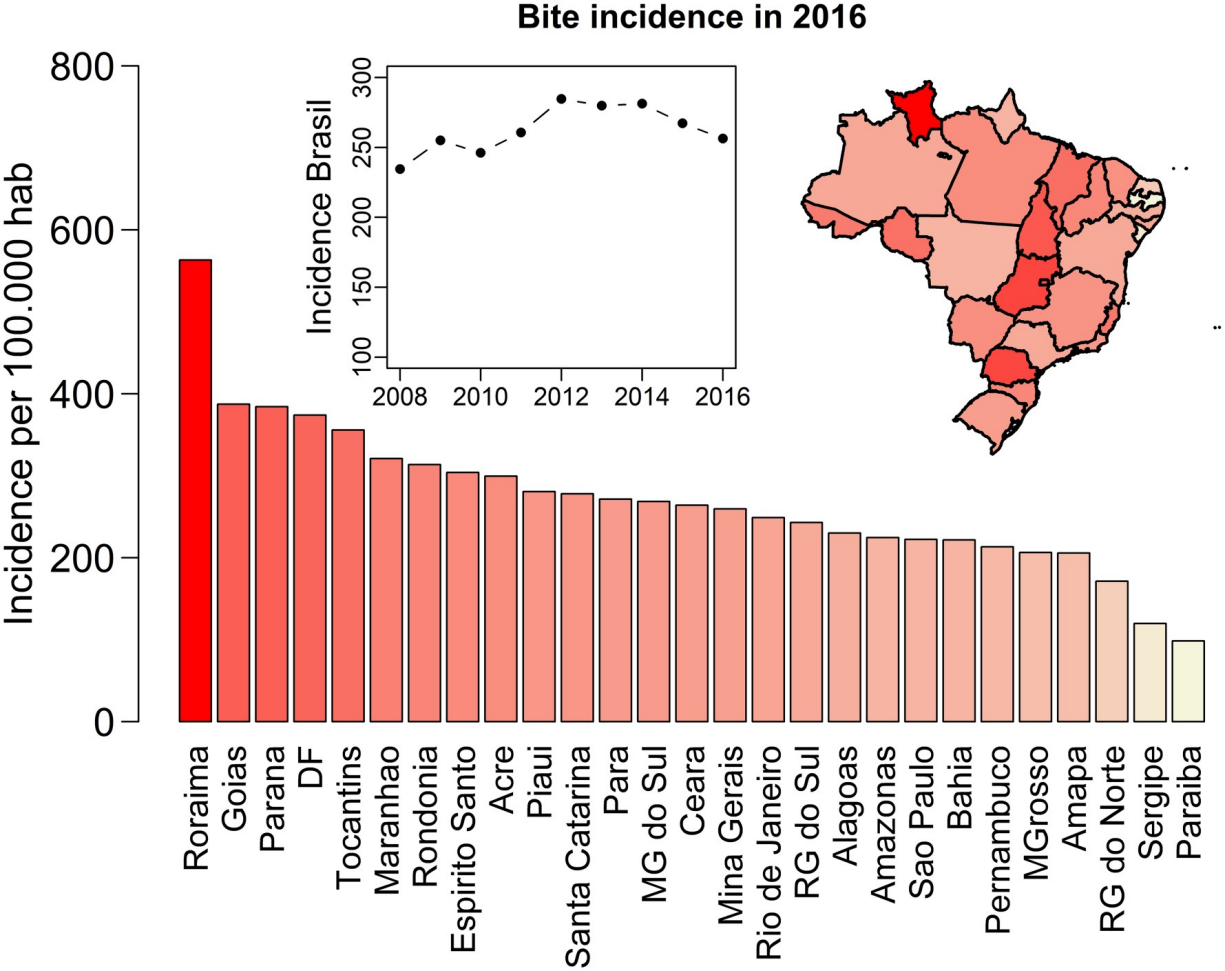
Bite incidence in each state of Brazil during 2016. The main bar plot shows the bite incidence (i.e. number of patients seeking health care after a bite per 100, 000 people) in each state during 2016. The map shows the same distribution in space with states coloured according to their bite incidence. The inner plot shows the country’s average bite incidence per year from 2008 to 2016. Country and state maps were obtained from the GADM (http://www.gadm.org/) database using the *getData* function from the *raster* package of R.

The incidence of patients seeking heath care following a bite (noted here as ‘bite incidence’) in Brazil remained relatively stable from 2008 to 2016, with an average of 257 patients/100 000 population (range: 231–280). Within that range, an increase was observed from 2008 (230 patients/100 000) to 2012 (279 patients/100 000), followed by a decline until 2016 (252 patients/100 000) (Fig 2). Annual bite incidence is, however, very variable per state with a maximum of 544 bites/100 000 population in the state of Roraima and a minimum of 97 bites/100 000 population in Sergipe in 2016 (Fig 2). There was no correlation between bite incidence in a state and either the state’s average income (Spearman’s correlation test, Rho = 0.20, p-value = 0.31) in 2016, the state’s number of houses with at least one dog (Spearman’s correlation test, Rho = -0.18, p-value = 0.37) in 2013, nor the state’s percentage of houses with at least one (Spearman’s correlation test, Rho = 0.19, p-value = 0.33).

**Fig 3:**
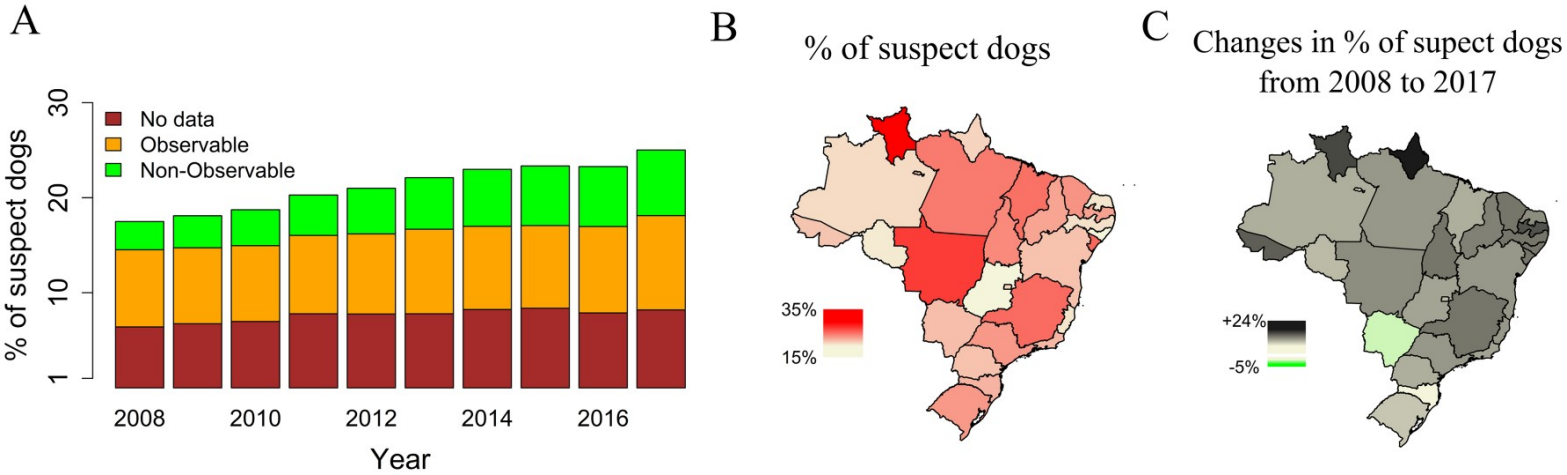
Changes in the percentage of dogs assessed as suspect for rabies between 2008 and 2017. A) Percentage of reports including a dog assessed as ‘suspect’ for rabies (i.e. assessed by a health worker as ‘rabies suspicious’, ‘rabid’ or ‘dead/disappeared’) per year in Brazil. The relative percentage of dogs assessed as ‘non-observable’ for a 10-day observation period (green), ‘observable’ (colour) or ‘no-data’ (brown) are shown within each bar. (B) Map showing the average percentage of dogs reported as ‘suspect’ for rabies annually per state between 2008 and 2017. (C) Map showing changes in this percentage from 2008 to 2017 across states. Positive numbers illustrate an increase in the percentage of suspect dogs. Country and state maps were obtained from the GADM (http://www.gadm.org/) database using the *getData* function from the *raster* package of R.

The percentage of patients bitten by dogs classified as suspect by health workers (including categories ‘rabies suspicious’, ‘rabid’ or ‘dead/disappeared’ in the SINAN form), which are expected to result in the use of PEP, increased from 17% in 2008 to 25% in 2017 (Fig 3A). This increase was mainly due to an increase in the number of dogs that were categorized as ‘non-observable’, which more than doubled (3% to 7%) during this period. Among dog bites, the absolute number and the relative proportion due to suspect rabid dogs varied between states (Fig 3B) and over time from 2008 until 2017 (Fig 3C). For example, the maximum proportion of bites due to suspect dogs was observed in Roraima state (35%) and the minimum proportion in Alagoas (15%). Likewise, the difference in the percentage of bites from suspect dogs between 2008 and 2017 was the highest in Roraima, which increased by 24%, whereas the only reductions were observed in the states of Santa Catarina (-6%) and Mato Grosso do Sul (-0.4%).

### Comparison between confirmed rabid dogs in SINAN and SIRVERA

**Fig 4:**
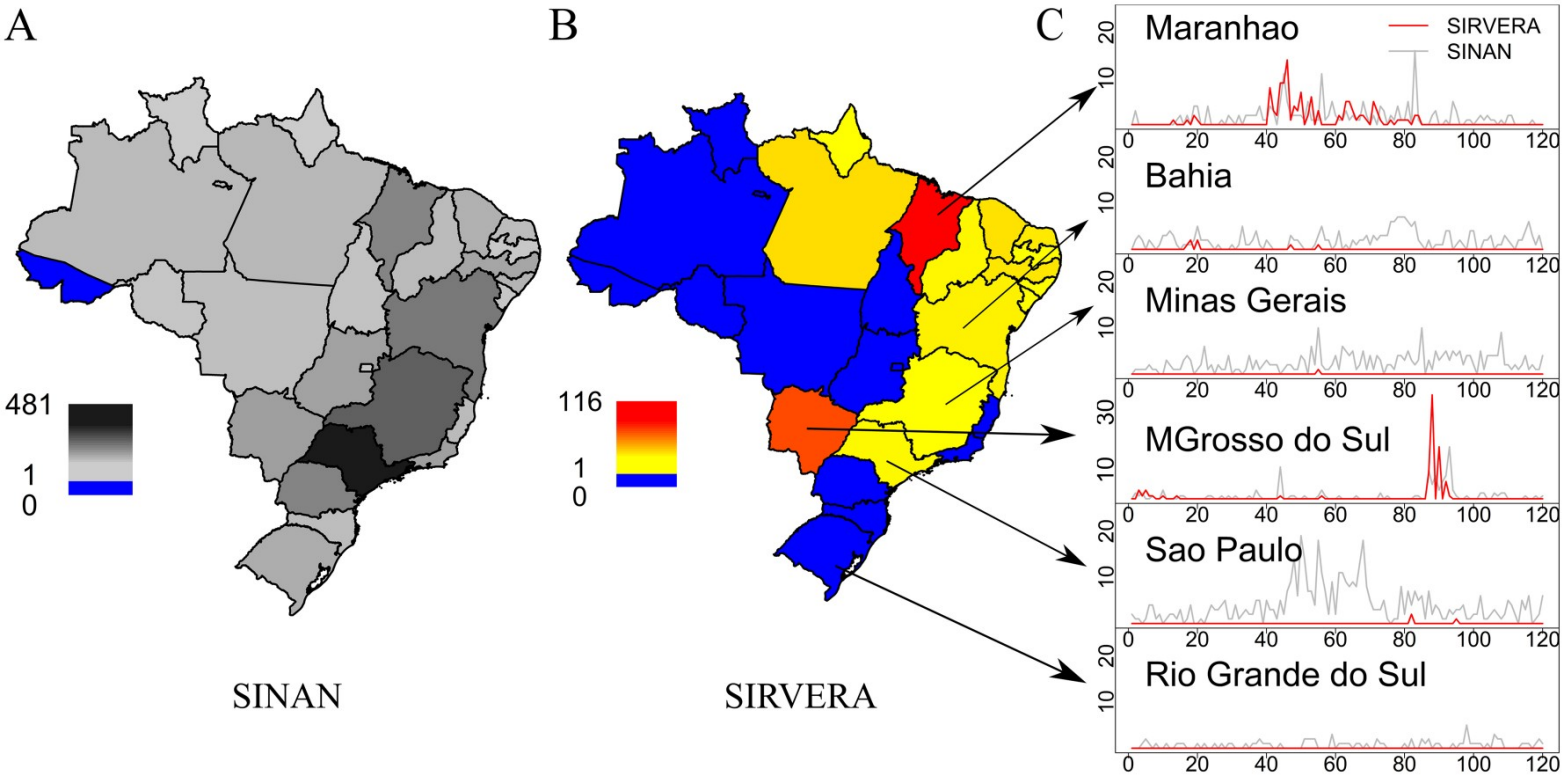
Comparison between rabid dogs reported to SINAN and SIRVERA. A) Map showing the number of dogs reported as ‘laboratory confirmed’ for rabies in the SINAN database per state between 2008 and 2017. B) Map showing the number of dogs reported as ‘laboratory confirmed’ for rabies in the SIRVERA database per state during the same time period. States coloured in blue did not report a positive case. C) Each plot shows the monthly number of positive cases in both SINAN and SIRVERA databases for selected districts from January 2008 to December 2017. Country and state maps were obtained from the GADM (http://www.gadm.org/) database using the *getData* function from the *raster* package of R.

The total number of dogs reported as ‘laboratory confirmed rabies’ in SINAN was much higher (2482 dogs) than in the SIRVERA database (269 dogs) for the period of 2008–2017. More than 2000 reports in the SINAN data could therefore be considered as ‘false positives’, assuming these were not verified for inclusion in SIRVERA. Out of the 27 states in Brazil, 26 reported at least one lab-positive rabid dog in SINAN whereas over the same period only 14 states reported confirmed rabid dogs in SIRVERA (Fig 4A and 4B). States reporting the most positive rabid dogs also differed between SINAN and SIRVERA. In SINAN, larger more populous states such as Sao Paulo and Minas Gerais reported the most positive dogs with 481 and 296 respectively (Fig 4A), while from SIRVERA the states of Maranhão and Mato Grosso do Sul reported the most positive dogs with 116 and 83 respectively (Fig 4B). On a monthly basis, trends in rabies-positive dogs were similar in SINAN and SIRVERA for states with known foci of rabies transmission, such as Maranhão (2011–2014) and Mato Grosso do Sul (2015) (Fig 4C). However, in states with very few or no rabies cases identified in SIRVERA, such as Bahia, Minas Gerais, Sao Paulo and Rio Grande do Sul, monthly data on positive dogs from SINAN showed very different temporal trends, with peaks in cases that were not evident in SIRVERA (Fig 4C).

### PEP use and cost savings under new guidelines

**Fig 5:**
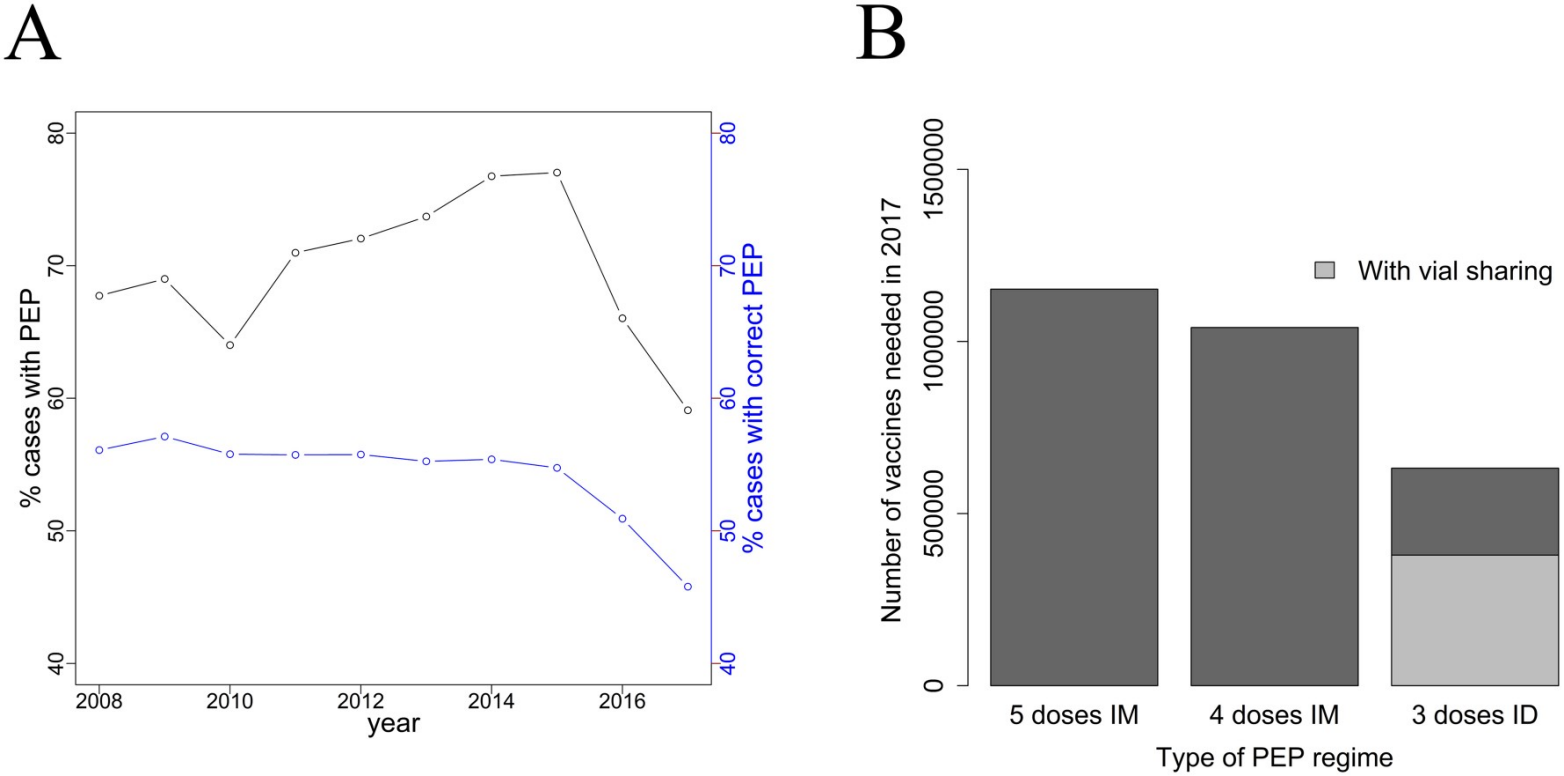
Percentage of appropriate PEP use and number of vaccine doses under different scenarios of PEP regimes. A) Percentage of cases receiving PEP from the total number of patients attended each year in Brazil (black line) and percentage of cases receiving the ‘appropriate’ recommended PEP according to the Ministry of Health guidelines (blue line). B) Estimated number of vaccine doses required to appropriately attend all patients during 2017 under three hypothetical scenarios: (i) the 2016 protocol in Brazil comprising 5 doses of vaccine according to the intramuscular (IM) Essen regimen (ii) the updated 2017 Brazilian guidelines using the updated IM Essen regimen with 4 doses and (iii) the latest WHO position comprising 3 intradermal (ID) doses. The light grey bar within the latest WHO regime corresponds to the number of vaccines needed in a scenario where vials would be shared completely between patients.

Based on SINAN, there was an increase in the percentage of patients requiring PEP (either vaccines or vaccine and serum) from 2008 (67%) to 2015 (77%). In the last two years, however, there has been a reduction with 59% of patients receiving PEP in 2017, which coincides with a vaccination shortage experienced by Brazil in 2015 (Fig 5A). This reduction was observed in 19 out of 27 states. Based on the PEP guidelines from the Ministry of Health used from 2008–2017, we estimated the percentage of patients that received appropriate PEP. The percentage of patients receiving appropriate PEP remained relatively stable at approximately 56% from 2008 (56%) to 2015 (54%). However, appropriate PEP administration declined in the last two years to 45% in 2017 (Fig 5A). Most of the incorrect PEP use in 2017 was due to either an under-use of vaccines or serum in high-risk bites that required PEP (37% of total PEP) or an unnecessary use of vaccines and serum in low-risk bites that did not require PEP (8% of total PEP). Under-use following a national vaccine shortage in 2015 increased from 25% to 37% (2017) of total PEP.

We used patient presentations in 2017 as a baseline from which to estimate vaccine requirements under different hypothetical PEP regimens. We estimated that 1,152,057 doses of vaccines were needed for appropriate PEP administration using the 5 dose IM Essen regimen that was recommended up to 2017 across Brazil. In comparison, we estimated that 1,040,444 doses would be needed under the 4 dose IM updated Essen regimen recommended in the updated MoH guidelines in 2017 and the WHO (Fig 5C). If Brazil implemented the latest WHO recommendations (e.g. 3 ID doses for complete prophylaxis), the number of required doses without vial sharing would be reduced to 631,835 (39% reduction from the 4 dose IM regime), and with vial sharing to 379,101 (64% reduction). Considering a price of 10 USD for each 0.5 mL vaccine vial, the WHO recommendations without vial sharing (6.31M USD) would save 4.08 M USD in vaccine costs compared to the current 4 IM guidelines (10.40M USD). Under the hypothetical scenario with complete vial sharing (3.79M USD), savings of up to 6.61M USD in vaccine costs could be made. In practice considerable vial sharing would be expected, especially in urban clinics with high throughput of patients, and so savings of between 4.08 M USD and 6.61M USD would be expected.

## Discussion

An effective national surveillance system is essential to detect remaining cases of dog-mediated rabies and to ensure that patients bitten by dogs that pose a risk of rabies transmission are given appropriate PEP. Control programmes in Brazil have substantially reduced dog-mediated rabies transmission and human cases of dog-mediated rabies in the last decade. Despite this reduction in dog rabies, our results show that the number of patients presenting to health facilities due to bites by dogs have remained relatively constant in the country over the last decade. However, the number of bites by high-risk ‘suspect’ dogs indicating the need PEP have increased. This mismatch between rabies cases and suspicion of a rabid dog suggests a ‘fear’ of rabies by health workers that has contributed to a risk averse increase in PEP use over the last decade, with only half of patients being administered PEP appropriately. Following the human rabies vaccine shortage experienced in 2015, the percentage of patients receiving appropriate PEP declined. We do not know of any human rabies deaths from dog-mediated rabies as a result of this apparent misuse of PEP and suggest that more judicious use of PEP could be safely undertaken if health workers are appropriately trained in risk assessments of bitten patients and use available epidemiological data to evaluate the risk of dog rabies in their area. Our analysis also showed that switching to ID administration of rabies vaccines according to the latest WHO position could save millions of dollars to the Brazilian health system related to vaccine volume, although the implementation of such a switch requires further investigation.

The overall incidence of patients in Brazil seeking care due to dog bites (255 bites/ 100,000 people) was similar to estimates from Kenya (289 bites) [32] but higher than incidence in patients reported in Tanzania (12–120)[33], Uganda (40) [34,35] and Switzerland (180) [36]. Community-based surveys focusing on dog bites, not on patients seeking care, show a much larger bite incidence in India (1700–2500) [37] and the USA (300–1000) [38]. Although bite incidence has remained relatively stable over the last decade in Brazil, bite incidence varies more than 6-fold between states, resulting in an uneven burden to state health centers that assess and treat patients. There was also a small increase up to 2012 followed by a decrease. Although the causes of this temporal variation remain unknown, changing dog populations, dog ownership patterns and access to health care could all have contributed to the observed decline after 2012. For example, large densely populated states such as São Paulo have a relatively low bite incidence but a high absolute number of patient presentations compared to less populous states such as Roraima that have a much higher incidence of bite patients. Because most of these bites come from healthy dogs, investigation of the factors underpinning these differences is warranted.

In our study, we did not find any correlation between bite incidence and each state’s average income nor the number (or percentage of houses) with at least one dog, suggesting that bite incidence was not necessarily explained by socio-economic status nor the overall dog population. A more detailed analysis at the municipality level could reveal local drivers associated with bite incidence such as the dog population (and human:dog ratio), the percentage of free-roaming dogs or people’s attitudes to dogs and their care. For example, some states might have a disproportionately higher population of free-roaming dogs or dog breeds that are more prone to bite as well as a higher proportion of at-risk populations (e.g. young males under 20 years old [26,39]. A particularly high bite incidence was observed in the state of Roraima, which neighbours with Venezuela. Attention should be given to improve measures in this state, since bites pose a considerable public health burden even in the absence of rabies and information on rabies in Venezuela is currently limited. In Roraima, no rabies cases have been reported to SIRVERA in the last decade but there is little active surveillance, which could contribute to a slow public health response if rabies were to emerge.

Despite a substantial reduction in dog-positive cases in the last decade, our analysis shows that the percentage of bites from dogs that are considered ‘suspect’ for rabies (i.e. showing symptoms, death or disappear) has increased. Most of the increase is due to dogs that were reported to not be observable. This increase is also very variable across states, with some states such as Roraima showing an increase of over 30%. An increase in the percentage of non-observable dogs could arise from an increase in bites from free-roaming dogs or a decreased in bites from owned (observable) dogs due to more responsible pet ownership. Alternatively, this could be due to increased unwillingness of health workers to perform dog follow up and the recommended 10-day observation and to directly prescribe PEP to avoid risks related to PEP underuse. Current data cannot resolve either of these hypotheses which requires further research. Similarly, about a third of reports from suspect dogs do not have data entered on whether the dog is observable or not, which highlights the need for better training of health workers in this crucial step that should determine PEP administration.

If dogs are not properly observed and assessed following a bite, this will increase the chance that genuinely rabid dogs will not be identified, which could result in further dog-to-dog transmission, or even failure to detect bat-to-dog transmission without any emergency response or prevention measures. In states with endemic transmission of rabies in the last five years, such as Maranhão and Mato Grosso do Sul, data from SINAN matches reasonably well with the positive cases reported to SIRVERA, suggesting that SINAN could be used effectively to enhance rabies surveillance. However, hundreds of ‘false positives’ were observed in other states such as São Paulo, Bahia or Minas Gerais. ‘False positives’ could arise if health workers accidentally mis-record data on SINAN forms and this data is not further checked. Alternatively, mistakes could be made to justify the use of PEP if health workers are not confident in recommending appropriate PEP. Discriminating between these hypotheses will require further work on the factors contributing to the recording of the SINAN data by health workers. We hypothesize that in states with endemic rabies healthcare workers may be more aware of risks because they have received specific and recent training on rabies by the Ministry of Health and PANAFTOSA following outbreaks and thus take more care to record data. In contrast, in states where rabies has been absent for many years health care workers may become lax in recording because the repercussions of over- or under-prescription may not have immediately apparent ramifications. States such as São Paulo or Minas Gerais have also the largest human populations of Brazil and thus, even if the percentage of mistakes on recording SINAN data is the same as in other smaller states, it results in a larger number of ‘false positives’. However, maintaining vigilance in rabies-free states is important when the risk of importation from endemic areas remains. Overall, these results highlight the need to both improve and update health care worker’s knowledge on the signs of rabies in dogs by strengthening a ‘one health’ link with veterinarians. For example, a user-friendly mobile app could i) put in contact state’s health workers with veterinarians to improve dog assessments, ii) improve dog follow ups, and (iii) rapidly detect a ‘false positive’ or priority areas for educational campaigns on dog rabies and vaccination. ‘One health’ workshops performed annually in each state could also help in updating knowledge of health and veterinarian professionals on the current rabies situation, dog assessment, and best surveillance practices.

Inadequate training of health workers may also limit the appropriate administration of PEP according to the guidelines of the Brazilian MoH. We estimated that PEP use increased over the last decade to almost 80% of patients seeking healthcare in 2015, although only around 58% of patients were administered PEP appropriately according to guidelines. Despite an increase in overall PEP use until 2015, the main cause of inappropriate PEP was ‘under-use’ of vaccines or serum when required for high-risk bites. The vaccine shortage experienced in 2015 reduced PEP use for the following two years, but also reduced the appropriate administration to 45% in 2017, and ‘under-use’ increased by 12% from 2015 to 2017. This suggests that vaccine shortages resulted in inappropriate PEP use rather than a more rationalized use. Given that no human rabies cases of dog-mediated rabies were observed as a result of inappropriate PEP use as defined by current guidelines, this indicates that further improvements can be made in identifying the risk that a biting animal does have rabies. Updating health workers knowledge on both national and state guidelines for PEP administration is urgently required, particularly in northern states such as Roraima, where the turnover of health professionals is high.

One approach to reduce PEP costs in Brazil would be adoption of the latest WHO recommendations [29]. We estimate that in 2017, the use of the 1-week abridged ID regimen could have saved at least 4.08 M USD only on vaccine volume. This reduction is much higher than changing to the 4 dose IM regimen recommended by the Brazilian MoH and the WHO. Reductions in vaccine volume related to adopting WHO recommendations will come with a cost of training health workers to use this technique and replace the current vial/transport system of PEP. Cost reduction of vaccines could outweigh possible obstacles associated with changing to ID, but a cost-benefit analysis should evaluate whether cost reductions could outweigh costs associated with training. Several countries (Philippines, Sri Lanka, Bhutan, several states in India, Bangladesh, and Madagascar) with a high number of people attending health care after a bite have successfully switched from IM to ID vaccination [40]. Furthermore, ID vaccination is already undertaken in several Brazilian states as pre-exposure prophylaxis of professionals working in at-risk laboratories or activities. Many health workers therefore already have experience of ID vaccination with the BCG vaccine for tuberculosis disease and more recently with fractionated dosing of yellow fever vaccine. A more detailed analysis of SINAN data at the municipality level could provide evidence of which health centers would have the highest levels of dose sharing based on patient throughput and identify small health centers where vial sharing is not possible due to a low number of patients seeking health care each day. The economic savings associated with a change in PEP administration could be used, for example, to improve activities of rabies surveillance and prophylaxis in a large population of Brazil, including people exposed to vampire bat bites.

Our analysis of the SINAN data allows several conclusions to be drawn about potential for improvements to surveillance and PEP administration at the state and national level, however we acknowledge several limitations. First, the SINAN dataset includes a small but unknown number of ‘duplicate’ records, corresponding to patients accessing more than one health centre and generating more than one SINAN form. We expect this percentage to be low and not to bias results at the state level. Second, errors on data recording, which are likely generating many of the rabies-positive dogs in the database, can mislead the assessment of whether PEP was appropriately administered. For example, health workers can mis-record the ‘rabid dog’ field in the form manually or when transcribing data from handwriting to digital format. These errors, adding ‘false positives’ to the dataset, will prevail if not checked by local or national authorities responsible for SINAN. In our analysis, although we reported and excluded missing data and attempted to minimize the influence of data inconsistencies, inaccuracies will undoubtedly still be present. Third, SINAN does not capture patients that seek private health care following a dog bite or patients who are bitten that do not seek health care. Therefore, our estimates of bite incidence, although high, are likely an underestimate of the true burden of dog bites, although more than 70% of patients seek emergency healthcare within the public SUS system [41,42]. Fourth, reports of rabid dogs to SIRVERA likely underestimated the actual number of rabid dogs in Brazil. This is mainly due to a non-automatic transmission of SINAN data to SIRVERA and a lack of data uploading from states or national levels. We stress the need to improve partnerships between national and regional authorities for a more coordinated effort to eliminate canine rabies in the continent. However, if rabies is circulating in dogs within a state, we expect that at least occasionally cases will be reported to SIRVERA because canine rabies is a mandatory notifiable disease in Brazil, dog rabies cases are routinely monitored and other species infected with rabies (e.g. bats) are detected through passive surveillance [1,43,44]. Detection capacity will ultimately depend on laboratory capacity, which remains variable between states and countries of Latin America [1]. Fifth, we did not obtain data from the last three months of 2017 from all states because the data, requested in 2018, was not yet entered to the national database. This shows that months of delays can limit timely control measures based on data from SINAN. Finally, data on the number of doses administered, although in the SINAN form, is not filled electronically to the main national system, which reduces our ability to estimate both appropriate PEP and potential for cost savings. Previous studies at the state level in Brazil have shown that 8–55% of patients do not complete the PEP prescribed and 70% of PEP courses are interrupted due to withdrawal of patients [45,46]. Overall, these limitations reinforce the need for improving the accuracy of SINAN so that it can be used as an efficient passive surveillance tool for dog rabies.

In summary, this study demonstrated an increasing and uneven burden related to the prevention of dog-mediated rabies to the Brazilian health care system, despite a major reduction in disease transmission throughout the country. Our results highlight that improving the training of health workers, automating the data recording system to identify and require correction of errors, and changing PEP administration protocols could substantially reduce healthcare costs. Focusing on Brazil as an example, this study calls for improving passive surveillance and strengthening ‘One health’ links between health workers and veterinarians in order to move forward towards the elimination of dog-mediated rabies across Latin America. Better use of PEP and enhanced surveillance could enable more efficient and effective allocation of resources towards other emerging public health problems such as PrEP and PEP for vampire bat rabies prevention.

## Supporting information

S1 Table Variables extracted from each SINAN form. (XLSX)

S2 Table Brazilian Ministry of Health guidelines for canine rabies prophylaxis. (XLSX)
